# A novel biosensor for the ultrasensitive detection of the lncRNA biomarker MALAT1 in non-small cell lung cancer

**DOI:** 10.1038/s41598-021-83244-7

**Published:** 2021-02-11

**Authors:** Mei Chen, Dongming Wu, Shihua Tu, Chaoyin Yang, DeJie Chen, Ying Xu

**Affiliations:** 1 Clinical Laboratory, Clinical Medical College and The First Affiliated Hospital of Chengdu Medical College, Chengdu, 610500 Sichuan People’s Republic of China; 2 School of Bioscience and Technology, Chengdu Medical College, Chengdu, 610500 Sichuan People’s Republic of China

**Keywords:** Biochemistry, Biotechnology, Nanoscience and technology

## Abstract

Long non-coding RNAs (lncRNAs) have been proposed as diagnostic biomarkers for the screening of non-small cell lung cancer and monitoring disease progression. Accordingly, new, rapid, and cost-effective lncRNA biosensors that can be used clinically are urgently needed. Herein, a novel effective and ultrasensitive electrochemical biosensor was developed based on a gold nanocage coupled with an amidated multi-walled carbon nanotube (Au NCs/MWCNT-NH_2_)-decorated screen-printed carbon electrode (SPCE). Because of its large surface area, superior conductivity, and excellent biocompatibility, this SPCE Au NCs/MWCNT-NH_2_ lncRNA biosensor showed a wide linear range (10^–7^–10^–14^ M) and low limit of detection limit (42.8 fM) coupled with satisfactory selectivity and stability. Compared to traditional RT-PCR, the proposed method exhibits acceptable stability, good selectivity, is simpler to operate, has faster detection, and uses less costly raw materials. In summary, this biosensor may be a powerful tool for detecting lncRNAs for efficient clinical prognosis and cancer diagnosis.

## Introduction

Non-small cell lung cancer (NSCLC) is the most common type of lung cancer, accounting for 85% of all newly diagnosed cases. As most patients are diagnosed at an advanced stage, their prognosis remains poor despite recent progress in chemotherapeutic treatments^1^. Detection, particularly at an early stage, is the most important challenge in cancer treatment and may save millions of lives^2^. Thus, non-invasive or minimally invasive tests for early cancer detection must be developed. “Liquid biopsy,” which evaluates molecular markers in biological fluids such as the blood, may be useful for cancer detection. This non-invasive diagnostic technique enables rapid sampling and real-time repeatable detection.

Measurement of the levels of long non-coding RNAs (lncRNAs) in the blood has gained attention in recent years. lncRNAs are RNA molecules with a length > 200 nucleotides and play important regulatory roles in various physiological processes^3, 4^. Blood-borne lncRNAs can be obtained through minimally invasive procedures and thus have potential as biomarkers for clinical diagnostic and prognostic purposes^5^. For example, studies have shown that the circulating levels of the lncRNA metastasis-associated lung adenocarcinoma transcript 1 (MALAT1) are considerably higher in plasma samples from patients with NSCLC than in those from healthy controls^6, 7^. Therefore, MALAT1 may be useful as a diagnostic biomarker for screening and monitoring the progression of NSCLC.

Despite considerable progress in detecting lncRNAs, few approaches can be applied clinically. LncRNAs are large and present in the blood at low levels, making sensitive analysis difficult^8, 9^. LncRNAs are commonly detected by microarray-based methods and RNA-sequencing; however, these strategies generally involve amplification steps and require cumbersome sample pretreatment procedures, large amounts of serum, and costly instrumentation, limiting their application in clinical diagnosis^10^. Thus, real-time polymerase chain reaction (RT-PCR) is the gold standard for measuring RNA levels and is widely used to quantify small/medium RNA targets with high sensitivity^11, 12^. However, RT-PCR also requires expensive devices with controlled thermal cycling and is highly sensitive to contamination by genomic DNA^13, 14^. Thus, a new, simple, rapid, and cost-effective class of lncRNA sensors that can be used with clinical samples are urgently needed.

Electrochemical biosensors based on functional nanomaterials may provide an alternative, highly sensitive, fast, and convenient method for detecting cancer biomarkers^15, 16^. Moreover, using a screen-printed carbon electrode (SPCE), which is small and inexpensive, as the working electrode offers the advantages of simple operation, portability, and miniaturization. However, few studies have examined the potential of SPCE-based biosensors for detecting lncRNAs. To improve the sensitivity of electrochemical RNA biosensors, signal amplification strategies based on functional nanocomposites have been proposed^17^, wherein large amounts of electrochemical mediators and natural micromolecules are loaded onto the nanomaterials^18, 19^.

Gold nanoparticles (Au NPs) are highly efficient nanomaterial-based catalysts that facilitate a large number of reactions, including the reduction of oxygen and alkene hydrogenation, and have been utilized in numerous electrochemical bioanalyses^20, 21^. Given the success of NPs, considerable effort has recently been devoted to fabricating hollow metallic spheres with a larger specific surface area than their solid counterparts to achieve better catalytic effects^22^. Au nanocages (Au NCs) are innovative materials in the field of biosensing because of their hollow interior and porous walls^23, 24^.

Recently, well-aligned, multi-walled carbon nanotubes (MWCNTs) were developed to improve the sensitivity of electrochemical detection^25, 26^. MWCNTs are ideal materials for electrochemical biosensing because of their high electrical conductivity, large length-diameter ratio, large surface area, and excellent mechanical strength^27^. MWCNTs can be modified by incorporating hydrophilic primary amines to produce MWCNTs-NH_2_ which show good dispersion in water and low cytotoxicity, and can easily be loaded with other nanomaterials or biomolecules^28, 29^. Although Au NCs and MWCNTs have been used in electronic devices and supercapacitors, the combination of Au NCs with MWCNTs-NH_2_ has not been explored. Furthermore, their use in the electrochemical detection of lncRNAs has not been investigated.

**Scheme 1:**
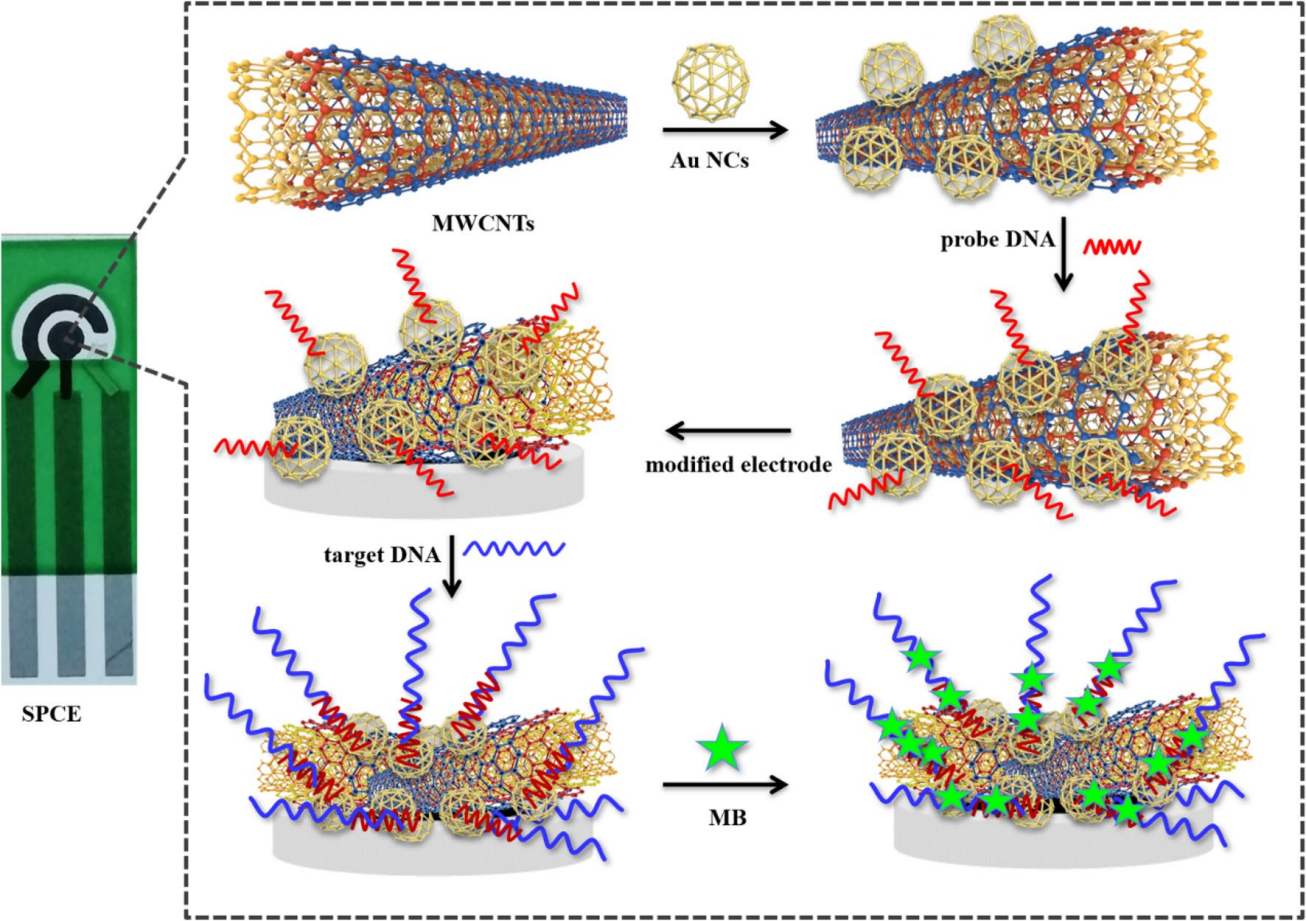
Schematic representation of the SPCE electrochemical DNA biosensor.

In this study, an effective, ultrasensitive SPCE-based electrochemical biosensor using an Au NCs/MWCNT-NH_2_ nanostructure was used to produce a sensitive, inexpensive assay for detecting MALAT1 (Scheme 1). We found that Au NCs/MWCNTs-NH_2_ can be loaded with a probe capable of specifically detecting MALAT1 and that this binding event alters the electrochemical properties of the biosensor. Furthermore, using SPCE as the working electrode in this biosensor enabled miniaturization, portability, and low cost. Compared to traditional RT-PCR, the proposed biosensor exhibited high sensitivity, acceptable stability, and good selectivity. This SPCE-based electrochemical strategy is also economical and rapid, and represents a quantitative alternative for detecting lncRNA levels in the clinic.

## Materials and methods

### Apparatus and materials

Electrochemical measurements were performed using a CHI 600E electrochemical workstation with anSPCE system composed of a carbon working electrode, carbon counter electrode, and Ag/AgCl reference electrode. Morphological characterization was performed using an FEI Nova 400 field-emission scanning electron microscope (Hillsboro, OR, USA) and ZEISS LIBRA 200 transmission electron microscope (Oberkochen Germany). X-ray photoelectron spectroscopy was performed using an ESCALAB 250 photoelectron spectrometer (Thermo-VG Scientific, Waltham, MA, USA). MWCNTs were purchased from XF Nano Technology Co., Ltd. (Nanjing, China). Gold chloride, methylene blue, Tris–HCl, polyvinylpyrrolidone, ethylene-diaminetetraacetic acid disodium salt (Na_2_-EDTA), dicyclohexyl carbodiimide, and DNA hybridization buffer were purchased from Sigma-Aldrich (St. Louis, MO, USA). All other reagents were purchased from Zicheng Yibo Biochemical Co., Ltd. (Chengdu, China), and all chemicals were of analytical grade. Probe RNA, MALAT1, HOTAIR, H19, and miRNA126 sequences were synthesized and purified by Sangon Biological Engineering Tech. Co., Ltd. (Chengdu, China). All oligonucleotide stock solutions were prepared in a Tris–EDTA buffer (10 mM Tris, 1 mM EDTA, pH 7.4). The specific sequences were as follows:

- MALAT1: 5′-ACTTAACAATTTTGTGTAATAAAAATGGAGAAGCTCT-3′
- HOTAIR: 5′-TTTTATGCATAAATAAAGTTTTACATGTGGTGAATAT-3
- H19: 5′-GAGCCCTGGACTCATCATCAATAAACACTGTTACAGC-3
- miRNA126: 5′-CAU UAU UAC UUU UGGUAC-3′
- Three-base mismatched MALAT1 target DNA (3MT):
- 5′-ACTTAACAACTTTGTGTAATAAGAATGGAGATGCTCT-3′
- One-base mismatched MALAT1 target DNA (1MT):
- 5′-ACTTAACAATTTTGTGTAATAAAACTGGAGAAGCTCT-3′

### Preparation of Au NCs

Au NCs were synthesized as previously described^30, 31^. Briefly, 1 mg mL^−1^ polyvinylpyrrolidone was added to 5 mL of deionized water to produce a homogeneous solution, to which 500 mL of Ag nanocubes (3 nM) was added before being boiled for 10 min. Gold chloride (0.5 mM) was added to the flask at a rate of 45 mL h^−1^, and the solution was refluxed for a further 30 min until the color of the reaction mixture was stable. After cooling to 25 °C, the sample was centrifuged, washed with saturated NaCl solution to remove AgCl, and washed three times with water to remove polyvinylpyrrolidone and NaCl.

### Synthesis of Au NCs/MWCNTs-NH_2_

First, an oxidizing acid was used to introduce active carboxyl groups at the ports or defects in MWCNTs to synthesize carboxyl-modified MWCNTs (MWCNTs-COOH)^28^. To achieve this, MWCNTs (500 mg) and concentrated nitric acid (68%, 150 mL) were mixed and stirred at 70 °C for 4 h. After cooling to 25 °C, the mixture was filtered through cellulose filter membranes (450-nm) and then cleaned with ionized water. The products were dried in a vacuum freeze dryer at 40 °C for 12 h. Next, MWCNTs-NH_2_ were prepared via a condensation reaction between the –COOH and –NH_2_ groups by adding 0.4 g dicyclohexyl carbodiimide to a solution of 20 mg MWCNTs-COOH in 2.5 mL ethylenediamine. The reaction mixture was stirred for 96 h at 120 °C, centrifuged, and filtered through microporous filter membranes. Finally, the resulting MWCNTs-NH_2_ were obtained after washing with deionized water and drying in a vacuum at 35 °C. The MWCNTs-NH_2_ were dispersed in a solution of synthesized Au NCs by stirring for 10 h and centrifuging gently at 2000 r/min for 5 min. The supernatant was changed to assess whether the Au NC solution was present in slight excess. The Au NCs/MWCNTs-NH_2_ were obtained by centrifugation and dried under vacuum at 35 °C.

### Fabrication of lncRNA biosensor

The surface of the SPCE was cleaned with anhydrous ethanol and then coated with 5 µL of the 2 mg mL^−1^ Au NCs/MWCNT-NH_2_ suspension that had been air-dried at 25 °C. The MWCNT-NH_2_ and Au NC-modified SPCEs were prepared in the same manner. To immobilize the 5′-thiolated RNA as the capture probe, 5 µL of lncRNA solution (1 × 10^−8^ M) was drop-cast over the modified SPCE and incubated for 30 min at 30 °C. The probe RNA was immobilized on the Au NCs/MWCNTs-NH_2_ via a strong Au–S bond and electrostatic interactions with the positively charged Au NCs^32^.

### Hybridization and electrochemical measurements

Hybridization was performed by immersing the probe RNA-modified SPCE in 0.01 M PBS (pH 7.0) containing various concentrations of the target lncRNA at 30 °C for 50 min. The hybridized SPCE was rinsed with PBS to remove any nonspecifically adsorbed lncRNAs, incubated in a 20 µM methylene blue solution for 10 min, and then washed with sodium dodecyl sulfate solution. Cyclic voltammetry (CV) was performed with cycling at a potential from − 0.2 to + 0.6 V and scan rate of 100 mV/s. Electrochemical impedance spectra (EIS) were determined over a frequency range of 10^−1^–10^5^ Hz in [Fe(CN)_6_]^3−^/^4−^ (1.0 mM) containing 0.1 M KCl. The electrochemical response was measured by differential pulse voltammetry (DPV) in 5.0 M PBS (pH 7.0), and scanning was performed from − 0.1 to + 0.3 V with a sweeping rate of 50 mV s^−1^. To evaluate the clinical applicability of the biosensor, all methods were carried out in accordance with relevant guidelines and regulations (Declaration of Helsinki). All experimental protocols were approved by the Medical Ethics Committee of Chengdu Medical College and informed consent was obtained from all subjects or, if subjects were under 18 years of age, from a parent and/or legal guardian.

## Results and discussion

### Characterization of Au NCs/MWCNT-NH_2_ composite

**Figure 1:**
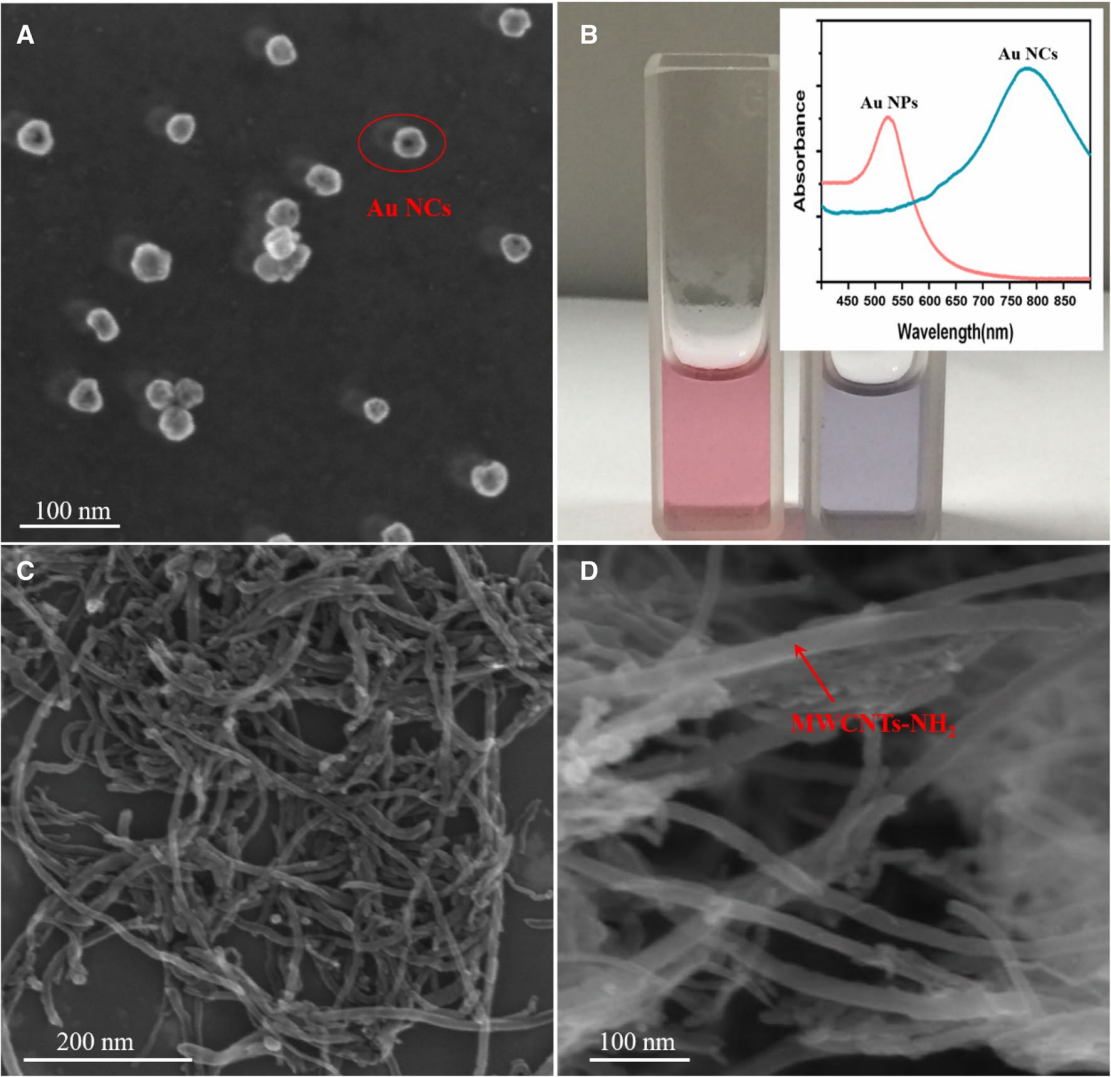
(**A**) SEM images of Au NCs. (**B**) Photograph and UV absorption spectra of Au NPs and Au NCs. (**C**, **D**) SEM images of MWCNT-NH_2_ with different magnification.

Scanning electron microscopy (SEM) images showed that Au NCs were well-dispersed and highly uniform (Fig. 1A). The Au-NPs were orange-purple, whereas Au NCs were blue. The ultraviolet absorption spectrogram is shown in Fig. 1B. SEM observations revealed typical morphological features for the MWCNT-NH_2_, with an average length and diameter of approximately 15 and 20 μm, respectively (Fig. 1C,D). These results support that the Au NCs and MWCNT-NH_2_ were successfully synthesized.

**Figure 2:**
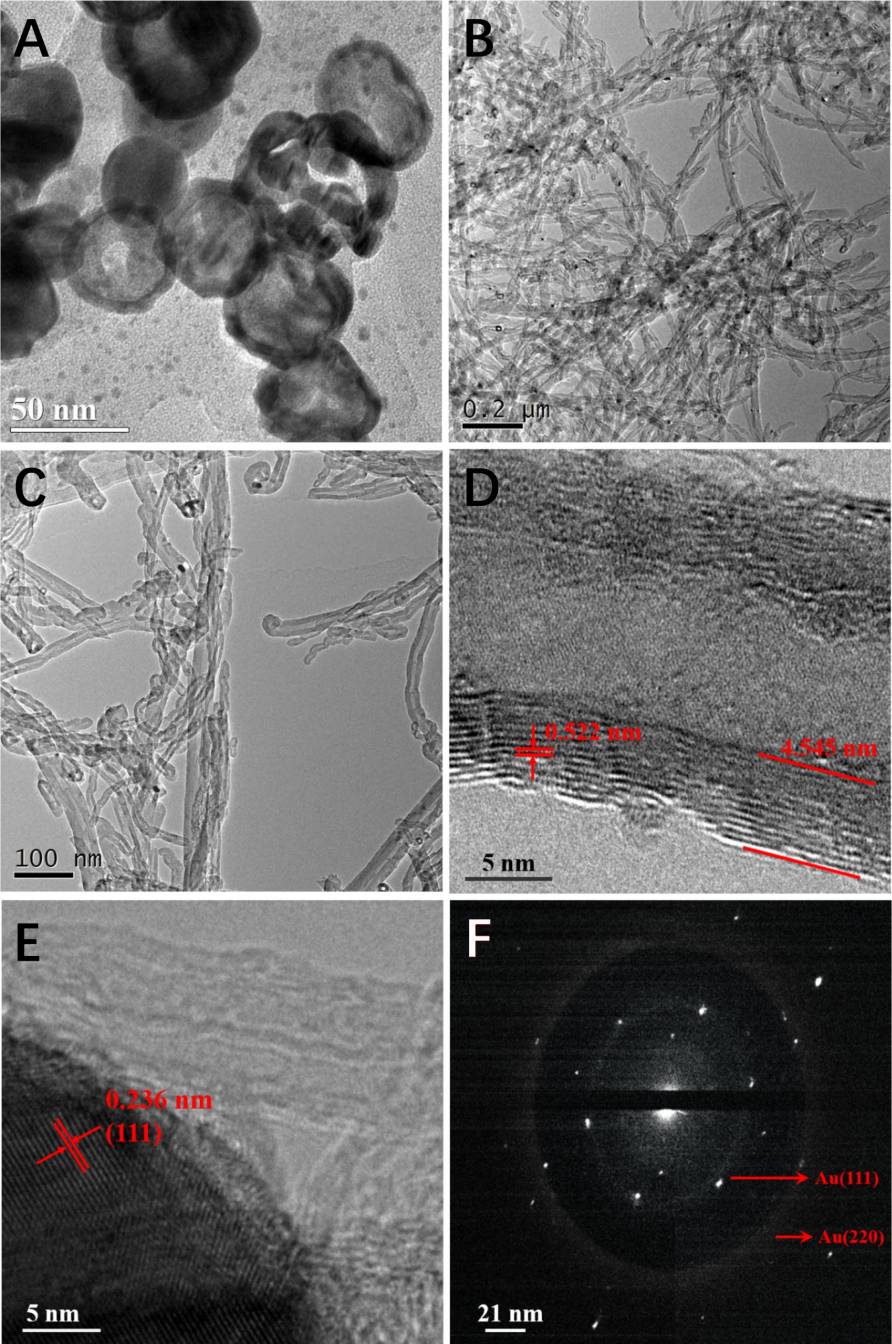
(**A**) TEM images of Au NCs. (**B**, **C**) TEM images of MWCNT-NH_2_ with different magnification. (**D**, **E**) High-resolution TEM images of Au NC/MWCNTs-NH_2_. (F) Selected area electron diffraction image of the Au NCs.

As determined by transmission electron microscopy (TEM), the Au NCs were around 40–50 nm in size and had hollow structures (Fig. 2A). After the MWCNTs-NH_2_ and Au NCs were mixed, large numbers of Au NCs were successfully linked to the surface of MWCNT-NH_2_ (Fig. 2B,C), demonstrating that Au NCs can attach to MWCNT-NH_2_. Moreover, the corresponding lattice fringes were visible in the high-resolution TEM images. These images showed that the MWCNT-NH_2_ wall was 4.545 nm thick and the fringe lattice was 0.522 nm thick, corresponding to the (111) crystal plane (Fig. 2D). The interplanar spacing was approximately 0.236 nm (Fig. 2E), supporting the d-spacing of the (111) lattice plane for Au. Furthermore, the electron diffraction pattern of small individual NPs had a ring-like area (Fig. 2F), with interplanar distances corresponding to the face-centered cubic phase of Au.

**Figure 3:**
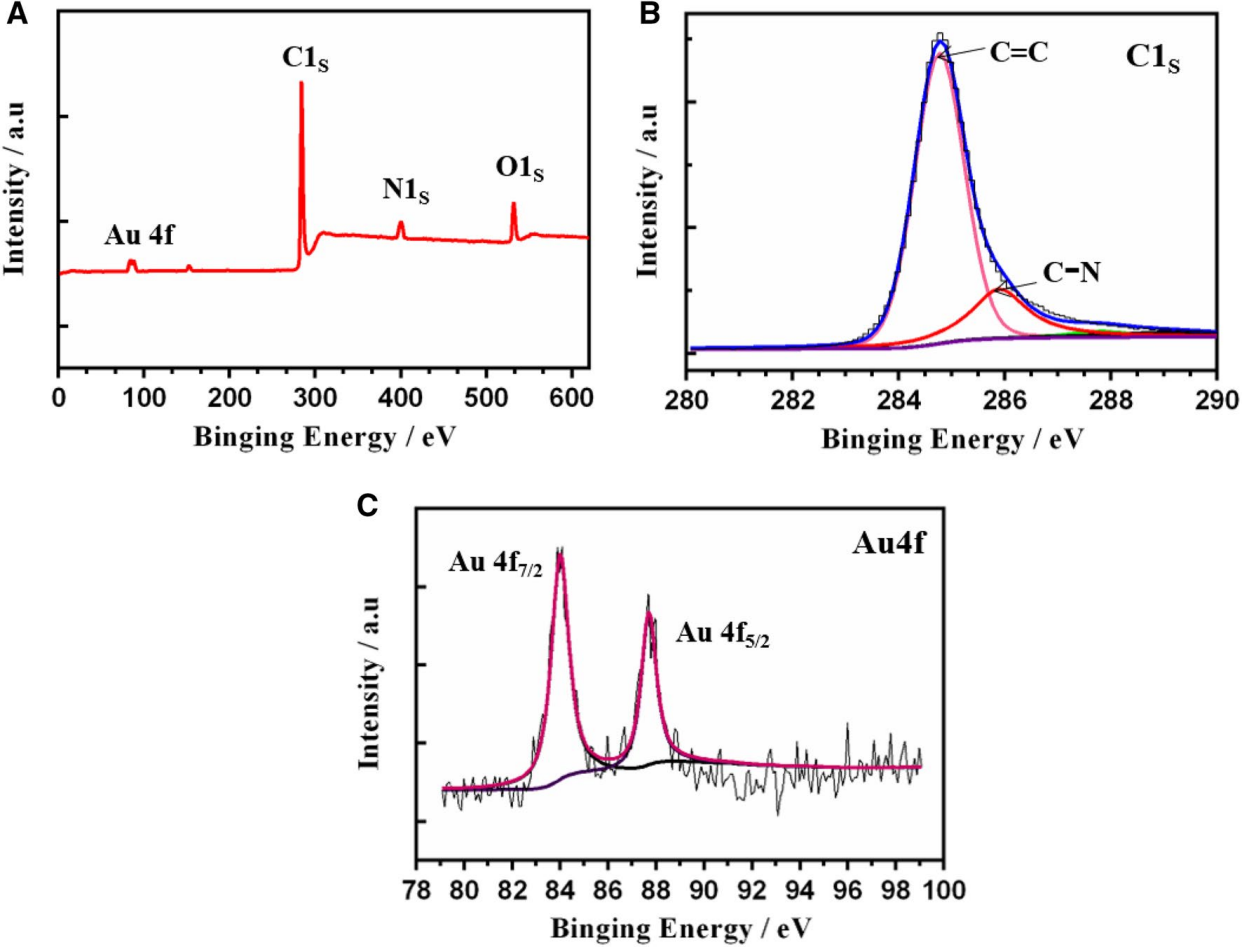
(**A**) X-ray photoelectron spectra of Au NC/MWCNTs-NH_2_. (**B**) High-resolution C 1 s and (**C**) Au 4f. narrow scan of Au NC/MWCNTs-NH_2_.

X-ray photoelectron spectroscopy was performed to analyze the surface chemical composition of the Au NCs/MWCNT-NH_2_. Peaks at 285.4, 398.6, 532.0, and 711 eV were associated with C1s, O1s, N1s, and Au 4f., respectively (Fig. 3A). In the C1s spectrum of Au NCs/MWCNT-NH_2_ (Fig. 3B), the absorption peaks at 284.6 and 285.0 eV corresponded to the sp2 and sp3 hybrid graphite-like structural carbons on MWNTs, respectively^33^. The peak at 286.6 eV corresponded to the C–NH binding energy, indicating the presence of –NH_2_ functional groups on the MWCNT-NH_2_ surface. Moreover, Au 4f. peaks were observed at 84.0 and 87.6 eV, which corresponded to Au 4f_7/2_ and 4f_5/2_, respectively (Fig. 3C), confirming the presence of metallic Au on the Au NCs/MWCNT-NH_2_ composites.

### Electrochemical behavior of modified electrodes

**Figure 4:**
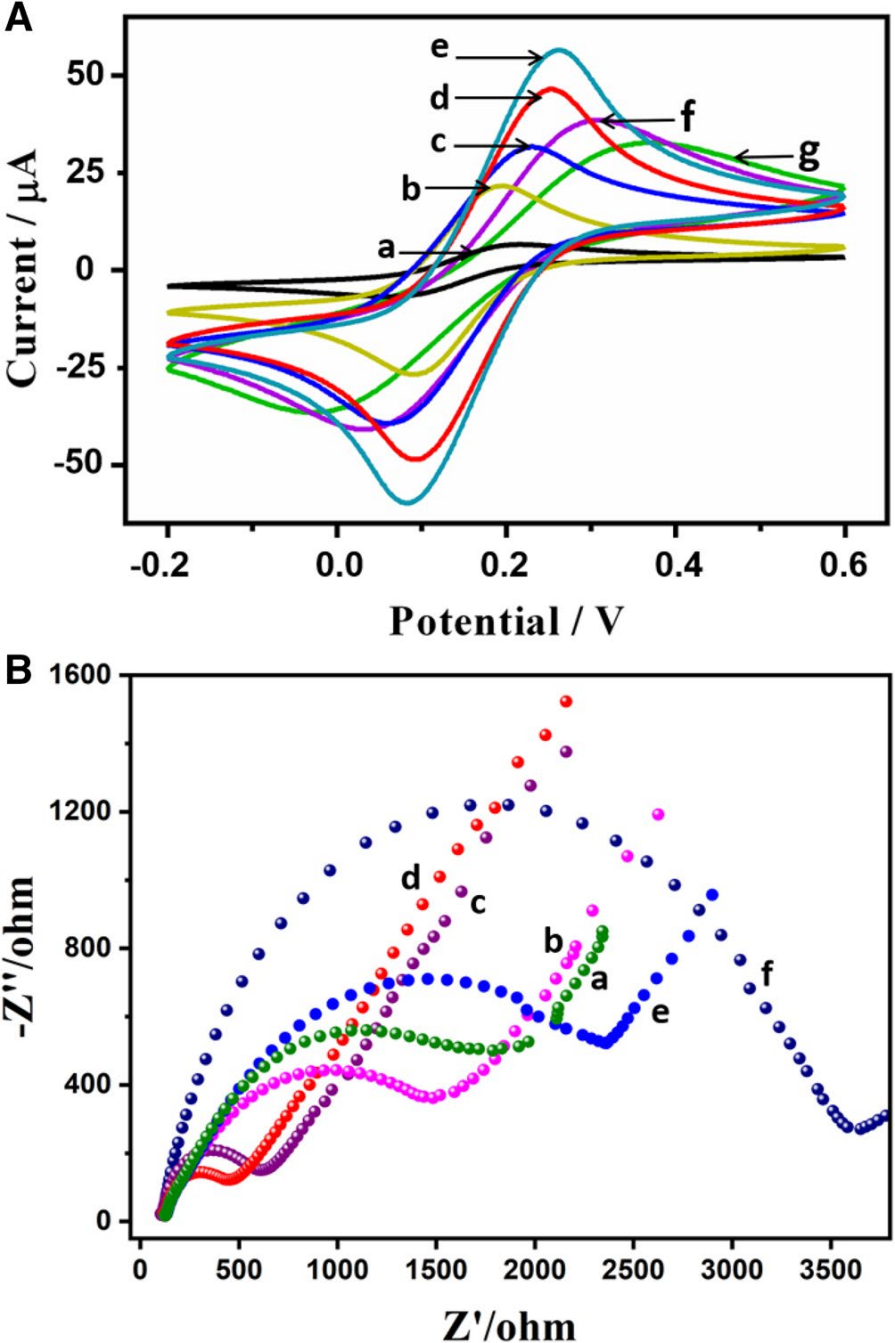
(**A**) Cyclic voltammograms of differentially modified electrodes in 1.0 mM Fe(CN)_6_^3−/4−^ and 0.1 M KCl solution at a scan rate of 100 mV/s. (a) Bare SPCE; (b) Au NPs/SPCE; (c) Au NCs/SPCE; (d) Au NP/MWCNTs-NH_2_/SPCE; (e) Au NC/MWCNTs-NH_2_/SPCE (f) probe-RNA/Au NC/MWCNTs-NH_2_ /SPCE; (g) hybridized-RNA/MWCNTs-NH_2_/SPCE. (**B**) Nyquist diagram of EIS corresponding to each step of the modification of the biosensor. (a) Bare SPCE; (b) Au NPs/SPCE; (c) Au NCs/SPCE; (d) Au NC/MWCNTs-NH_2_/SPCE; (e) probe-RNA/Au NC/MWCNTs-NH_2_/SPCE; (f) hybridized-RNA/MWCNTs-NH_2_ /SPCE. The inset displays the Randles circuit to fit the experimental EIS data.

CV is the most effective and convenient method for investigating electrochemical processes occurring at electrode interfaces. CV was performed on different modified electrodes in 1.0 mM [Fe (CN)_6_]^3−^/^4−^ solution containing 0.1 M KCl at a scan rate of 100 mV s^−1^ (Fig. 4A). Compared to bare SPCEs (curve a), Au-NPs (curve b), Au NCs (curve c), and Au-NP/MWCNTs-NH_2_ (curve d), the biosensor using Au NCs/MWCNTs-NH_2_ as a label exhibited a much greater electrochemical response (curve e). This phenomenon may be attributed to two factors: (1) novel hollow Au NCs provide a higher specific surface area, more exposed active sites, and therefore exhibit excellent electrocatalytic activity and (2) MWCNT-NH_2_ is an attractive support for Au NCs, resulting in a synergistic combination with strong electrocatalytic performance. Next, the capture probes were assembled on the surface of Au NCs/MWCNTs-NH_2_ (curve f) and hybridized with the target lncRNA (curve g). The peak current continuously decreased as the negatively charged RNA biomolecules blocked [Fe(CN)_6_]^3−/4−^ electron transfer. This noticeable decrease in the peak current indicated that the RNA biosensor had been constructed successfully.

EIS analysis is an effective method for investigating the electrochemical properties of electrodes after each stage of modification. When Au-NPs attached to the bare SPCE (Fig. 4B, curve a), the electron transfer resistance (Ret) decreased (curve b). After electrode modification with Au NCs, a lower Ret was observed (curve c), which we ascribed to the ability of the hollow structure to more effectively promote electron transfer. Au NCs/MWCNTs-NH_2_ showed the lowest Ret (curve d). When nonconducting RNA molecules were introduced and loaded onto the nanomaterial surface, the Ret increased significantly (curves e, f). Based on the consistency of the EIS data with the CV data, we had successfully fabricated the SPCE-based biosensor.

### Optimization of experimental conditions

A key issue is how the reaction solution pH affects the sensor response; therefore, we evaluated how the reaction solution pH affected the biosensor performance. Figure S1a shows a CV plot of the fully assembled biosensor exposed to 10 fM mL^−1^ MALAT1 in 2 mL of 0.1 M PBS at varying pH (6.0–8.5). The peaked reached a maximum at pH 7.0; therefore, PBS at a pH of 7.0 was used as the optimum working buffer in subsequent experiments. The effect of the time used for nucleic acid hybridization to the biosensor was also examined. The probe RNA/Au NCs/MWCNT-NH_2_/SPCE was incubated with lncRNA solution (100 pM) for 20, 40, 60, 80, and 100 min, and the CV was recorded (Fig. S1b). The reduction in peak current reached a maximum at an extended hybridization time of 80 min. The peak current then increased because of the strict effect of hybridization events. These data demonstrate that the hybridization reaction was mostly complete after 80 min; accordingly, we used this hybridization time in subsequent experiments.

### Analytical performance of lncRNA biosensor

**Figure 5:**
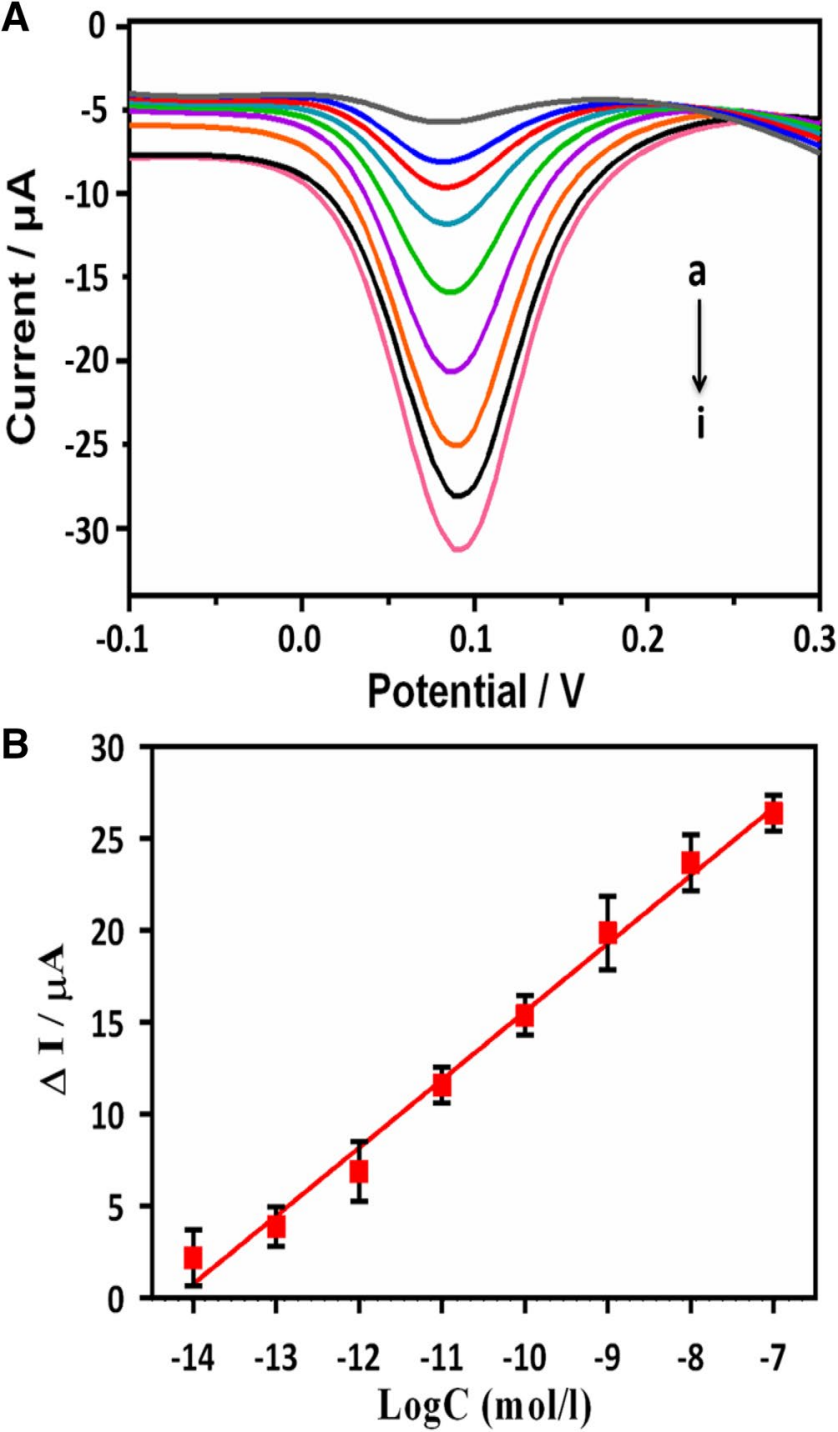
(**A**) DPV curves after hybridization with 0, 1.0 × 10^−14^ M, 1.0 × 10^−13^ M, 1.0 × 10^−12^ M, 1.0 × 10^−11^ M, 1.0 × 10^−10^ M, 1.0 × 10^−9^ M, 1.0 × 10^−8^ M, and 1.0 × 10^−7^ M target lncRNA MALAT1 (curves a–i, respectively). (**B**) The calibration plot of peak current versus the logarithm of the concentration of target lncRNA MALAT1; Error bars represent the standard deviations (n = 5).

To investigate the sensitivity of the proposed method for lncRNA detection, DPV was used to measure the electrochemical response after hybridizing an ss-DNA capture probe with the MALAT1 lncRNA target under optimized experimental conditions. The reduction in the peak current in response to the MALAT1 target increased linearly as the target concentration increased (10^–8^ to 10^–14^ M; Fig. 5). The regression equation was ΔI (μA) =  − 0.076logC–5.23 (R^2^ = 0.902). The detection limit was calculated as 42.8 fM with an S/N ratio of 3 (where S is the relative standard deviation of a blank solution). We also analyzed the performance of several types of biosensors that have been used to detect non-coding RNAs (Table 1). This analysis showed that the proposed biosensor had a satisfactory detection limit and linear range. The biosensor also had high sensitivity and a low limit-of-detection, which we attributed to the superior conductivity and large specific surface area of Au NCs/MWCNT-NH_2_s. Therefore, this biosensor is a promising platform for capturing large numbers of target lncRNAs and facilitating the electron transfer process. Moreover, this new, easy to use SPCE is a meaningful step in the field of biosensing.

**Table 1:** Comparison of linear ranges and detection limits of the different RNA biosensors.

Modified material and electrode	Techniques	Linear range (M)	Detection limit (M)	References
BND/BNF@GO/Au/HRP	DPV	0.01 nM–10 nM	0.247 pM	^34^
GR-COOH/hemin	DPV	0.5 pM–1.0 nM	170 fM	^35^
Au/Rh-HNP@ SWCNT	DPV	10 μM–1.0 pM	0.886 pM	^36^
AuNPs/GCE	DPV	0.01 nM–10 nM	2.57 pM	^37^
MB/MWCNT-COOH/GCE	DPV	0.1 pM–500.0 pM	84.3 fM	^38^
Au-rGO-PANI	DPV	0.1 pM–10 nM	50 fM	^39^
Au NCs/MWCNTs-NH_2_	DPV	0.01 pM–10.0 nM	42.8 fM	This work

### Biosensor specificity, reproducibility, and stability

**Figure 6:**
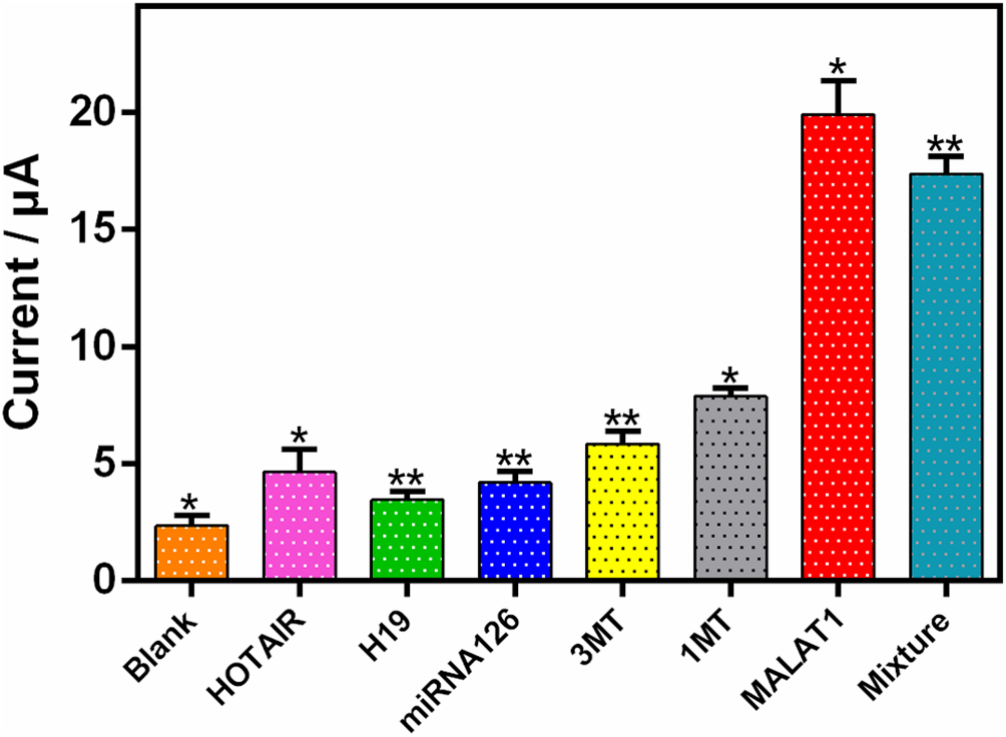
Specificity of the proposed biosensor: different types of sequences (HOTAIR, H19, miRNA126, single-base mismatch target (1MT), three-base mismatch target (3MT), MALAT1, and a MALAT1 mixture); Error bars are related to three independent measurements.

To investigate the specificity of the proposed method for assaying target lncRNAs, its selectivity was assessed using samples containing various potential interfering substances. These substances included HOTAIR, H19, miRNA126, and target lncRNA MALAT1 with a single-base mismatch target (1MT) or a three-base mismatch target (3MT), as well as a MALAT1 mixture. A 50-fold increase in selected RNAs (50 pM; HOTAIR, H19, and miRNA126) caused minimal current responses, similar to that in the blank test; however, the presence of a much lower concentration (1 pM) of the perfectly matched target MALAT1 lncRNA and its mixture resulted in a significantly higher current response (Fig. 6). Additionally, the peak current was higher with 1MT compared to 3MT. These results demonstrate that the proposed biosensor has ideal specificity for detecting lncRNAs and, therefore, great potential for clinical use.

To assess the reproducibility of the biosensor, five electrodes were prepared to detect lncRNA. The relative standard deviation of measurements was less than 4.6% (n = 5). In addition, the stability of the RNA biosensor was evaluated after storage for 14 days at 4 °C, with measurements taken every 2 days. The CV peak current of the RNA biosensor decreased gradually, with the final peak current retaining 88.54% of the initial current after 21 days. Therefore, the reproducibility and stability of the proposed biosensor were acceptable for detecting lncRNA.

### Analytical and clinical applicability of the biosensor

The practical feasibility of the proposed RNA SPCE biosensor for clinical application was investigated using the standard method of adding lncRNA analytes to human serum samples (n = 6). Using the optimal experimental conditions, the electrochemical signals from different serum samples were determined using DPV, and the recovery rate was calculated (see Table 2). Satisfactory recovery values of 94.5–102.32 were achieved, with a relative standard deviation of 1.92–4.17%. Therefore, the proposed biosensor may be useful for analytical detection of lncRNAs in the clinic.

**Table 2:** Determination of lncRNA MALAT1 in human serum samples (n = 6) with the proposed SPCE biosensor (Each value is the mean of five replicate experiments).

Serum sample	Add (nM)	Biosensing method (nM)	Recovery (%)	Relative standard deviation (%)
1	0.5	0.48	96.0	2.93
2	2.0	2.01	100.5	4.17
3	8.0	7.56	94. 5	1.92
4	15.0	14.84	98.93	2.13
5	25.0	24.09	96.36	1.58
6	50.0	51.16	102.32	3.26

## Conclusions

An ultrasensitive electrochemical biosensor was developed based on Au NCss-MWCNT-NH_2_-catalyzed amplification and successfully used for electrochemical detection of a spiked lncRNA in clinical serum samples. The Au NCs combined with MWCNTs-NH_2_ resulted in higher degrees of electron transfer and high electrochemical activity, which significantly enhanced signal detection. Because of the superior conductivity and large specific area of Au NCs/MWCNT-NH_2_, the new RNA biosensor had a wide linear range and low limit of detection for MALAT1 lncRNA, with satisfactory selectivity and stability. Moreover, the SPCE biosensor is easier to operate, has more accurate quantitation, has a faster detection method, and uses cheaper materials than traditional methods. However, multiple lncRNAs are often involved in the same tumor molecular mechanism, and combined detection of three lncRNAs can effectively improve the accuracy of cancer diagnosis. Thus, our future studies will involve joint detection of multiple lncRNAs.

## Supplementary Information

Supplementary Information
