# Supplementary material for: A novel biosensor for the ultrasensitive detection of the lncRNA biomarker MALAT1 in non-small cell lung cancer
Source: Sci Rep. 2021 Feb 11;11:3666. doi: 10.1038/s41598-021-83244-7 (PMC7878801; doi:10.1038/s41598-021-83244-7)
Supplement: Supplementary file 1 — Supplementary Information [file 41598_2021_83244_MOESM1_ESM.docx]

**Electronic Supplementary Material for**

**Scientific Reports**

# A novel biosensor for the ultrasensitive detection of the lncRNA biomarker MALAT1 in non-small cell lung cancer

**Mei Chen ^a^, Dongming Wu ^a^, Shihua Tu ^b^, Chaoyin Yang ^b^, DeJie Chen ^b^,** **Ying Xu ^a∗^**

^a^ Clinical Laboratory, Clinical Medical College and The First Affiliated Hospital of Chengdu Medical College, Chengdu, Sichuan 610500, P.R. China

^b^ School of Bioscience and Technology, Chengdu Medical College, Chengdu, Sichuan 610500, P.R. China

* Corresponding author: Ying Xu. Tel.: +862883016721; fax: +862862739582.

E-mail address: yingxu825@126.com (Y. Xu).

**Optimization of experimental conditions**

**
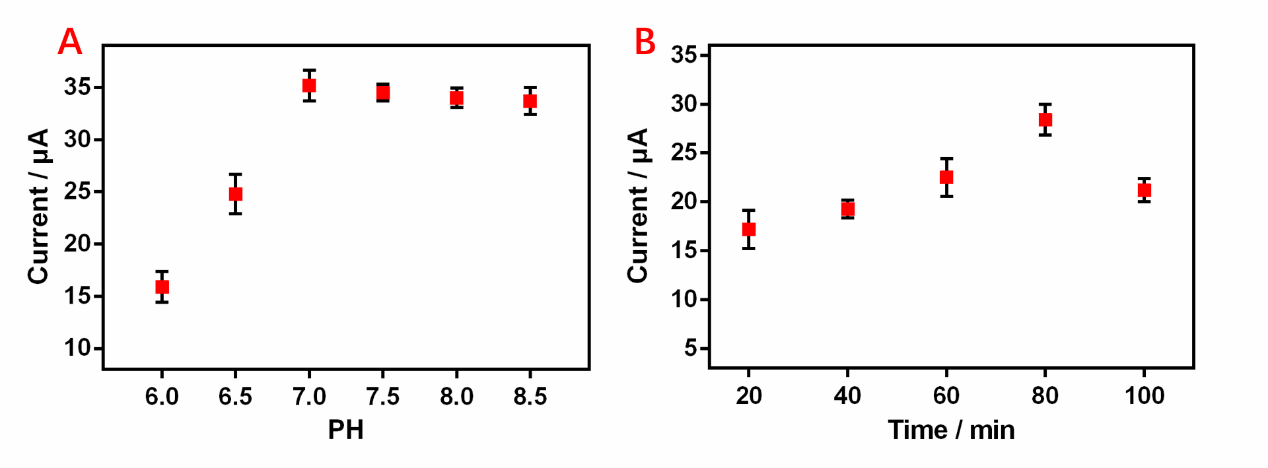
**

**Figure S1.** Optimization of the experimental conditions: Effects of (A) reaction solution pH, and (B) hybridization time on the lncRNA biosensor.
